# Gender bias in academic medicine: a resumé study

**DOI:** 10.1186/s12909-023-04192-6

**Published:** 2023-05-01

**Authors:** Elaine Burke, Elizabeth A. Heron, Martina Hennessy

**Affiliations:** School of Medicine, Trinity College Dublin, Dublin, Ireland

**Keywords:** Gender, Equality, Clinical academic training, Bias, Gender-concordant mentoring, Anonymisation

## Abstract

**Background:**

Minimising the effects of unconscious bias in selection for clinical academic training is essential to ensure that allocation of training posts is based on merit. We looked at the effect of anonymising applications to a training programme for junior doctors on the scores of the applications and on gender balance; and whether female candidates were more likely to seek gender-concordant mentors.

**Methods:**

Applications to the training programme were reviewed and scored independently by reviewers who received either an anonymised or named copy. Scores were compared using a paired t-test, and differences in scores compared by gender. The gender of named supervisors for male and female candidates was compared.

**Results:**

Scores of 101 applications were reviewed. When their identity was known, male candidates scored 1.72% higher and female candidates scored 0.74% higher, but these findings were not statistically significant (*p* value = 0.279 and 0.579). Following introduction of anonymisation, the proportion of successful female candidates increased from 27 to 46%. Female candidates were more likely to name a female supervisor compared to male (41% vs. 25% of supervisors).

**Conclusions:**

Anonymising applications did not significantly change scores, although gender balance improved. Gender-concordant mentoring initiatives should consider effects on mentors as well as mentees.

**Supplementary Information:**

The online version contains supplementary material available at 10.1186/s12909-023-04192-6.

## Introduction

Gender bias exists in all professions and academic medicine and surgery are no different. Women make up 8.8% of Fortune 500 CEOs [1], and only 28 of 193 UN Member States are led by women [2]. Women are under-represented at the highest levels in academia across all specialties. Even though women make up 46% of academic staff in third level institutions in Ireland, only 25% of full professors are female [3]. In the US, although 48% of medical school graduates are female and the proportion of female faculty is 41%, this is mostly at the lower levels and women make up only 25% of full professors and 18% of Department Chairs [4].

Reasons for gender imbalance in academic medicine and science have been well-described. Male candidates are considered significantly more hireable and more competent than an identical female candidate [5]. Female clinical academics are less likely to succeed when funding reviews focus on the investigator compared to reviews focussed solely on the scientific proposal [6]. Gender bias in the workplace creates additional barriers: 72% of female orthopaedic surgeons reported workplace conflict which they attributed to being female; 8% reported being forced out or leaving their job due to this conflict [7]. Even when women attain leadership roles, they can be stereotypically associated with specific types of work including institutional education and mentorship [8]. Gender equality is not only a social justice issue; gender balance has been shown to improve collaboration, patient outcomes and research productivity [9].

Implicit, or unconscious bias, occurs when automatic associations are made between members of a social group and a particular attribute or negative evaluation [10]. Healthcare professionals have been shown to manifest implicit bias associating women with family and men with career [11]. Institutions are advised to adopt strategies to minimise unconscious bias, including gender-blind reviews of job or grant applications [12, 13]. Gender-blinding is an attractive strategy because it removes the source of bias, and does not disadvantage other groups of candidates [14]. This strategy has been shown to be successful in fields such as astronomy and music [14, 15], but other studies in ophthalmology and molecular biology have found no effect [16, 17] and overall, evidence is limited [18]. We sought to investigate its effectiveness in recruitment to an early career training programme for junior doctors, the Academic Internship Track (AIT).

Launched in 2017, the AIT for Ireland is a one-year combined clinical academic training programme for junior doctors in their first postgraduate year. Junior doctors (interns) on the AIT are offered protected time to carry out a research project in a field of their choosing in addition to a bursary, dedicated study days and support from a named supervisor. Recruitment to the AIT involves submission of a CV and research proposal followed by an interview (Supplementary File 1). Following the first two years of recruitment to the AIT, a highly competitive process where applicants greatly outnumber the number of posts available, it was noticed that successful male candidates outnumbered female by approximately 3:1 for both years. In response to this finding, the AIT recruitment team decided to undertake a field experiment to investigate whether anonymisation would change how reviewers score candidates’ CVs and research proposals, and whether the effect would be different for male and female candidates.

### Same-gender supervisors

A lack of senior female role models resulting in fewer female mentors can be seen by junior staff to be an impediment to career development [19]. However, while studies show gender-specific mentoring is a popular intervention, there is little evidence that it is actually effective in improving gender balance [20], and an increase in the mentoring duties of senior female faculty at the expense of other activities such as research might represent a paradoxical barrier to career progression.

Candidates to the AIT identify supervisors themselves based on their field of interest; this is likely someone they have worked with in the past. In the second part of this resumé study, we compared the gender of supervisors indicated by male and female candidates to see if female candidates would be more likely than male to associate with a female supervisor.

## Materials and methods

### Study design

This was a resumé study evaluating data from the recruitment to the AIT in the 2019 and 2020 recruitment cycles. Administrative staff manually redacted names from each full application received. Full applications consist of a CV and a research proposal (see Supplemental File 1). Each application was reviewed by a minimum of three independent reviewers, each affiliated with one of the six medical schools in Ireland. Reviewers are all senior clinicians and academics. Three schools were randomly selected to receive anonymised applications; reviewers affiliated with the other three schools received non-anonymised or named applications. Each application was sent to reviewers from 3 different schools. Reviewers assign a score out of 100 for each application based on standardised criteria, with up to 60 marks available for the CV section and 40 marks for the research proposal. The highest scoring candidates following review are invited to the next stage of recruitment, an interview. The 24 highest ranking candidates following interviews are offered posts.

On receipt of the applications to be shortlisted, all reviewers were provided with a set of instructions and informed that some applications were anonymised in keeping with international recommendations on minimising implicit bias; included with the instructions was a link to the League of European Universities’ 2018 Advice paper on implicit bias in academia [13]. Reviewers received either all anonymised or all named applications.

Our outcome was application score, and the variable of interest was anonymisation. We evaluated candidates based on self-reported sex (Male/Female). We also collected data on the sex of supervisors named in the applications. During the recruitment process, we monitored the gender balance at each stage and compared it to gender balance at each stage in the preceding two years.

### Ethical considerations

This study was approved by the Trinity College Dublin School of Medicine’s Research Ethics Committee (September 2020, Application Number 20200502). Reviewers were contacted individually in November 2020, after completion of both recruitment processes, and asked to provide written consent that their scores could be used as part of the study.

### Data analysis

**Fig. 1:**
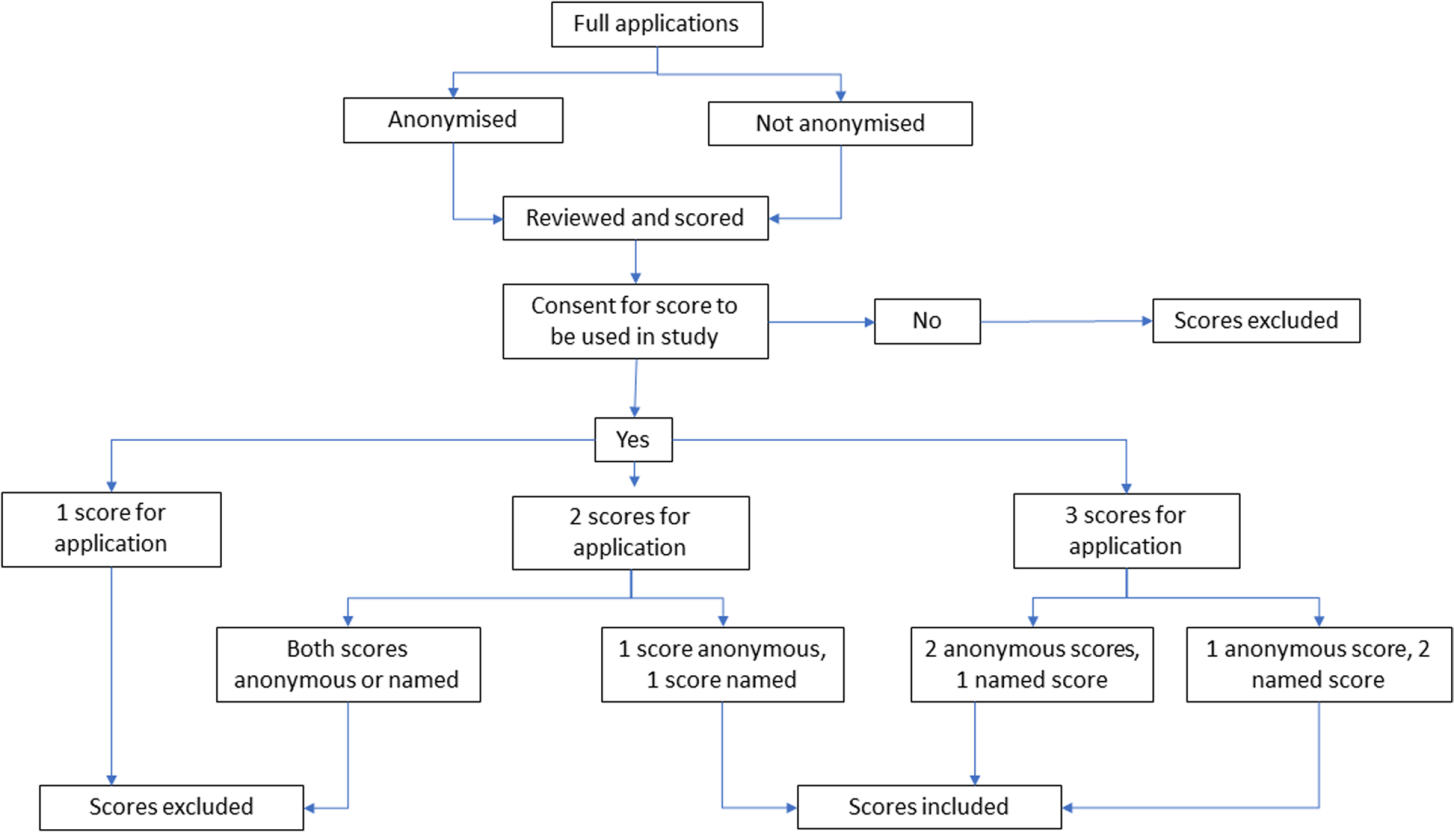
Flowchart explaining inclusion and exclusion criteria for data analysis

The analysis looked at the difference between scores when the application was named and when it was anonymous using a paired t-test. For an application to be included, it had to have been scored from both an anonymous and named perspective for comparison. Scores of reviewers who did not consent to participate were removed. Where there were three reviews, two of the scores came from either an anonymous or a named review and an average of these scores was taken. Where there were two reviews, only those who had both anonymous and named reviews were included (see Fig. 1).

As an additional measure to tackle unconscious bias in the recruitment process, in 2019 and 2020, interviewers were asked to undertake unconscious bias training (e.g., Living Equality and Diversity online training programme) prior to the interviews if they had not previously done so, and provided with links to their own institutions’ online unconscious bias training programmes. There is a degree of overlap between reviewers and interviewers, i.e., some, though not all, reviewers also interview candidates.

## Results

### Comparison of anonymised versus named applications shortlisting scores

#### Data summary: reviewers and applications

In 2018, most of the reviewers and interviewers were female (68.4% and 65% respectively). In 2019, there were fewer female reviewers (42.8%) but a similar number of female interviewers (66.7%).

Between 2019 and 2020, 32 reviewers out of 40 consented to participate in the study. No reviewers declined to allow their scores to be included, but eight did not respond to three emails. Reviewers were almost evenly divided between those who received anonymised applications (*n* = 17) and those who received named applications (*n* = 15). Of the 32 reviewers whose scores were included in the study, 12 were female and 20 were male (37.5% vs. 62.5%). The same schools received anonymous reviews for 2019 and 2020, so reviewers that partook in the process for both years only received either anonymous or named applications.

Scores of one hundred and thirty-seven applications were reviewed. Eleven applications were reviewed by one reviewer, 65 by two reviewers, and 61 by three reviewers. No applications had more than three reviews. One hundred and sixty-six reviews were anonymous (51.2%), 158 were named (48.8%). Seventy-one applicants (51.8%) were female, 66 (48.2%) were male. After averaging scores for applications which had two anonymous or two named scores, thirty-six applications were excluded because they had only one score. Scores which were excluded were those with only one review or with two reviews where both reviews were anonymous or named (Fig. 1).

### Comparison of anonymous and named application total scores

**Fig. 2:**
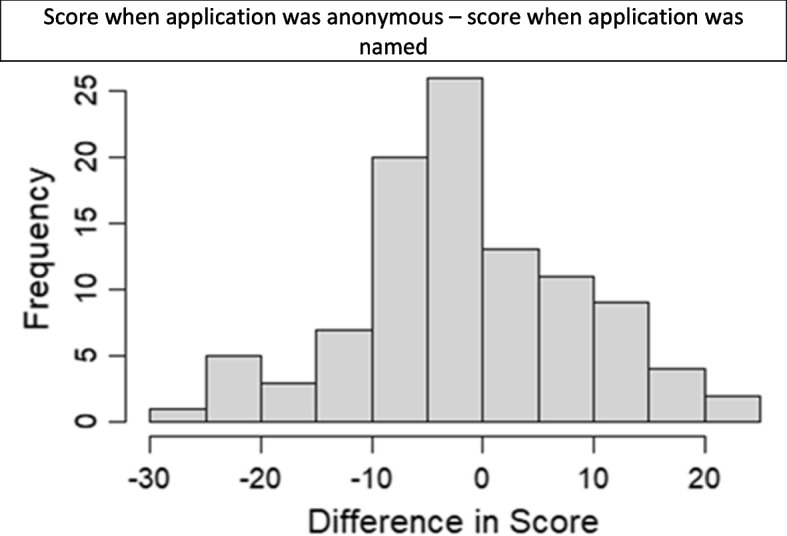
Histogram of difference between scores when the application was anonymous compared to named

Anonymous scores were compared to named scores using a paired t-test as there are two scores per application (Fig. 2). Applications scored an average of 1.19% lower when they were anonymous compared to named. This difference was not statistically significant. The 95% confidence interval for the difference between anonymous and named scores was [-3.19%, 0.82%], p-value 0.24.

### Comparison of anonymous and named application total scores with males and females considered separately

**Fig. 3:**
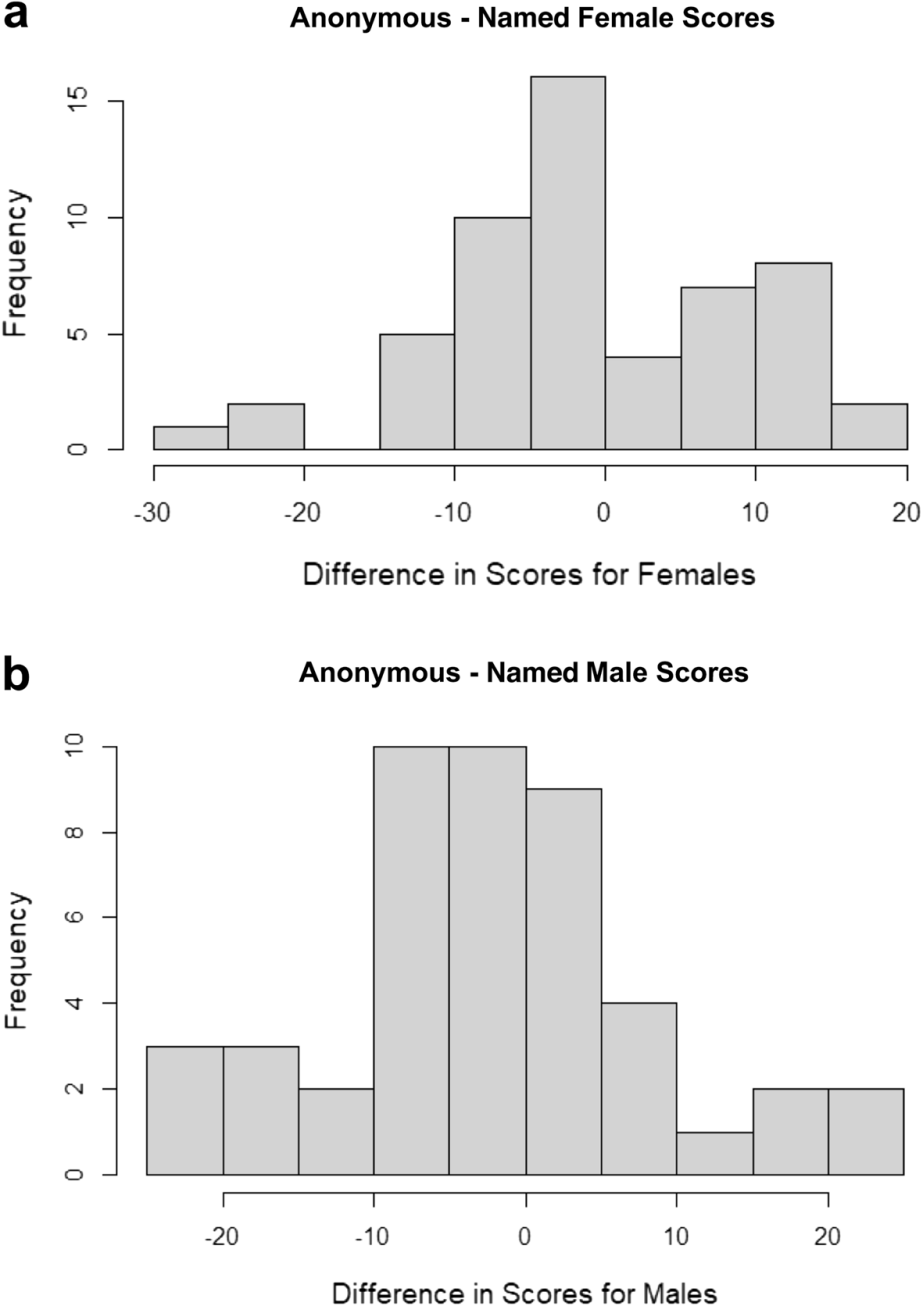
Histogram of differences between anonymous and named scores for female and male candidates. **a** Anonymous - Named Female Scores. **b** Anonymous - Named Male Scores

The female anonymous scores are on average 0.74% lower than female named application scores (Fig. 3). This difference is not statistically significant. 95% confidence interval for difference between anonymous and named scores for female candidates: [-3.40%, 1.92%], *p*-value = 0.58.

The male anonymous scores were on average 1.72% lower than male named scores. This difference is not statistically significant. 95% confidence interval for difference between anonymous and named scores for male candidates: [-4.87%, 1.44%], *p*-value = 0.28.

Please see Supplemental File 2 for distributions of average scores.

### Gender balance during recruitment to the AIT

Recruitment to the AIT runs in parallel to recruitment to standard internship. When standard internship recruitment opens, candidates are invited to indicate interest in the AIT by ticking a box. Those who tick the box are subsequently invited to submit full applications. Those who do not tick the box or decide not to submit a full application continue with the standard internship application and those whose AIT application is unsuccessful are returned to the standard process.

One thousand five hundred and forty-seven internship applicants have indicated their interest in the academic track at Stage 1 of the application process in the first four years of the AIT (2017–2020). Two hundred and seventy-nine full applications have been received, 186 interviews held, and 96 academic interns appointed (Table 1). Gender data (M/F) is available for all applicants.

**Table 1:** Number of applicants to academic track by stage of recruitment process

	**Number of Candidates by Stage of Process**			
**Year**	**Stage 1**	**Full Application**	**Interview**	**Post acceptance**
2017	413	80	48	24
2018	336	61	48	24
2019	352	56	48	24
2020	446	82	42	24
**Total**	**1547**	**279**	**186**	**96**

A similar number of male and female applicants have indicated interest in the academic track at Stage 1 over all four years. In the first two years, male candidates progressively outnumbered female candidates as the recruitment process continued: in 2017, 58.7% and in 2018, 57.4% of full applications received were from male candidates; 62.5% and 60.4% respectively of those offered an interview were male and male appointees outnumbered female by almost 3:1 for both years (Table 2).

**Table 2:**
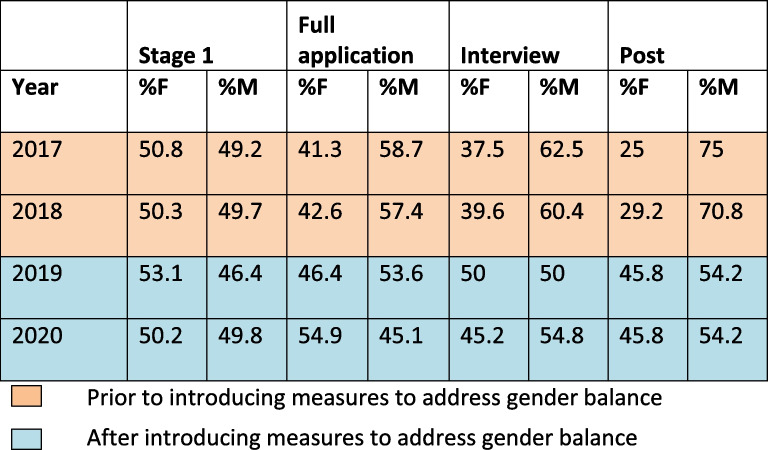
Ratio of male to female candidates during recruitment process by year

Following the introduction of anonymised applications and the request for interviewers to undertake unconscious bias training, ratios of male to female candidates remained similar throughout the process: 50% and 45.2% of those interviewed were female in 2019 and 2020, and there was almost 50:50 gender balance among appointees to the programme (Table 2).

### Gender of proposed supervisors

Two hundred and nine candidates submitting full applications (74.91%) identified one or more potential supervisors. Seventy candidates did not name a supervisor, 31 candidates named more than one supervisor:29 candidates named two, and 2 candidates named 3 supervisors.

Female candidates were on average more likely to name female investigators as supervisors compared to male candidates: 40.7% of supervisors named by female candidates were female compared to 25.2% of supervisors named by male candidates.

## Discussion

The first two years of recruitment to the academic internship track show a higher success rate for male candidates compared to female candidates: while both male and female students initiated the application process in similar numbers, successful male candidates outnumbered female by 3:1. Following the introduction of anonymised applications for some reviewers, gender balance improved. There were no other changes to the recruitment process, and changes in the distribution of male and female reviewers and interviewers does not explain this effect. However, anonymising applications did not make a statistically significant difference to scores.

Removing identifiers from applications seems like a reasonable approach to mitigating unconscious bias: reviewers cannot be biased if they are unaware of the applicant’s gender. However, anonymising job applications can have unpredictable effects, with some studies finding that the practice resulted in increasing women’s chances of interview, and others finding a reduced chance [21, 22]. An investigation of the effects of anonymisation on recruitment to an ophthalmology residency programme found no significant effect on applicant scores overall or specifically for female candidates [16]. When applications are anonymised, reviewers can seek implicit signals to categorise applicants according to gender, and in doing so, use stereotypes of employment patterns and communications styles, thus activating biases which the anonymisation procedure sought to suppress [21]. Our study is in keeping with these findings that anonymising applications alone may not be sufficient to tackle implicit bias.

One possible explanation for our finding is that our email communications with reviewers and interviewers which included information on mitigating gender bias might have constituted “cues for control” – a prompt to override prejudiced responses not in keeping with the individual’s beliefs and values (e.g., that people should be treated equally regardless of gender) [23]. Further investigation would be required to explore this hypothesis.

Female candidates were more likely than male candidates to identify a female supervisor for their project. Most candidates identify a supervisor that they have already built up a relationship with and have worked with in the past, so the supervisor may also be considered a mentor. The proportion of female supervisors selected by male candidates (25%) is the same as the known distribution of professorial posts among women in higher level institutions in Ireland (25%) [3] and internationally in academic medicine (25%) [4]. This might suggest that male candidates’ choice of supervisor is more in keeping with the proportion of senior faculty who are female, and potentially less influenced by the gender of the supervisor. This finding is supported in the literature: in a study of the impact of gender on mentor–mentee success in dermatology, < 40% of male participants (mentees) indicated that they would prefer a mentor of the same gender, while 80% of female participants reported that they would prefer a female mentor [24].

Female mentors can act as role models and share their experiences on issues specific to women e.g., balancing a career with the family responsibilities that usually fall to women. Protégés may also feel they have a greater connection and find it easier to communicate with same-gender mentors [24]. There are also a small number of female-dominated specialties (e.g. Child and adolescent psychiatry, public health) [25], and it is possible that female candidates are drawn more to these specialties than male candidates, hence will meet a higher proportion of potential supervisors who are female. However, with senior female faculty currently outnumbered approximately 3:1 in Ireland, and junior female faculty equalling junior male faculty in terms of numbers [3], same-gender mentoring risks over-burdening female faculty with the work of mentoring potentially at the expense of other work which would further their own careers, e.g., publication, creating a paradoxical barrier. Moving away from more traditional dyadic or 1:1 mentoring towards other models such as peer-mentoring, group mentoring or networking models may provide part of the solution because these models are less reliant on individual senior faculty member [26], although even these models typically require input from senior faculty e.g., facilitators in peer mentoring groups. Protégés benefit from mentorship regardless of gender concordance [24], so providing opportunities for mentoring relationships to develop without emphasising a need for same-gender mentoring is likely to benefit early career researchers while avoiding inequitable distribution of mentoring responsibilities.

### Limitations

Our study has the advantage of including data from real-life job applications and reviewer scores, however there are potential drawbacks to this approach. One limitation is that all the score differences in the analysis of the anonymisation process were treated as if they came from independent reviewers, whereas in reality, the same reviewer will have scored multiple applications. This creates a potential source of bias. It is difficult to fully anonymise academic applications where publications are included, furthermore gender-specific information can be inadvertently revealed in the CV section (e.g., captaincy of a camogie team, a women’s sport). Supervisor nominations were handled the same whether a candidate identified one or more supervisors, even when the genders of supervisors differed, creating another potential source of bias. Finally, implicit bias is an issue that doesn’t affect only women, there are recognised minority groups who are underrepresented in medicine (URiM). Due to a reliance on data gathered by the Irish Health Service Executive’s recruitment body, the Health Business Services (HBS), which currently only collects binary data relating to sex (M/F), it was not possible to analyse data related to other applicant characteristics including representation of groups who are URiM. Overlooking intersectionality is a cognitive pitfall which limits our ability to understand women’s experiences of discrimination [27].

### Outlook

Priorities for future work could include qualitative work exploring candidate’s choice of supervisor and how it may be influenced by gender; understanding barriers and incentives to applying for the AIT and how they might differ according to gender; and investigating the effect of anonymisation from the reviewer’s perspective, e.g., reviewer’s ability to correctly identify the gender of a candidate based on an anonymised CV, and their views on the effectiveness of anonymisation.

Even though we did not find that anonymisation made a significant difference to scoring, we decided to continue the practice under close monitoring because of the apparent effect on gender balance. At a minimum, receiving an anonymous application reminds reviewers of the risk of unconscious bias and may provide a cue for control. Given the lack of robust evidence for this approach, we recommend continuous monitoring and careful evaluation to avoid unintended consequences.

Measures recommended to enhance gender equality are often focussed on women, e.g., leadership or professional development training and gender-concordant mentoring [27]. Women are not the source of gender inequality, and even well-intentioned initiatives can paradoxically create barriers by demanding more of women’s time. We recommend investigation into solutions which tackle gender inequality at an organisational and societal level, giving appropriate recognition to intersectionality and the needs of groups who are URiM.

## Supplementary Information

**Additional file 1.**

**Additional file 2.**

## Data Availability

Datasets are available on reasonable request. Requests may be addressed to Dr Elaine Burke at burkee11@tcd.ie.
