# Supplementary material for: Gender bias in academic medicine: a resumé study
Source: BMC Med Educ. 2023 May 1;23:291. doi: 10.1186/s12909-023-04192-6 (PMC10152728; doi:10.1186/s12909-023-04192-6)

# Suplementary File 1: Additional information on the AIT

Taken from A Guide to Application and Appointment to Internship Training In Ireland Commencing July 2023. Full guide available at https://hbsrecruitmentservices.ie/wp-content/uploads/2022/10/2023-A-Guide-to-Application-and-Appointment-to-Intern-Training-in-Ireland-Stage-1-1.pdf


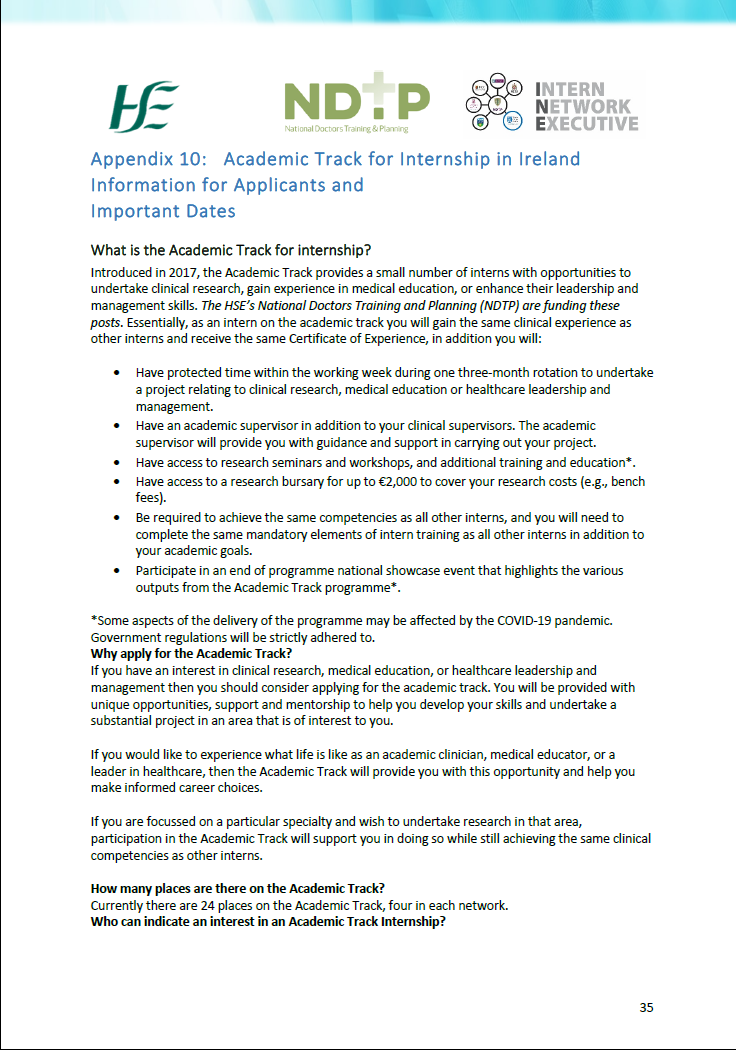


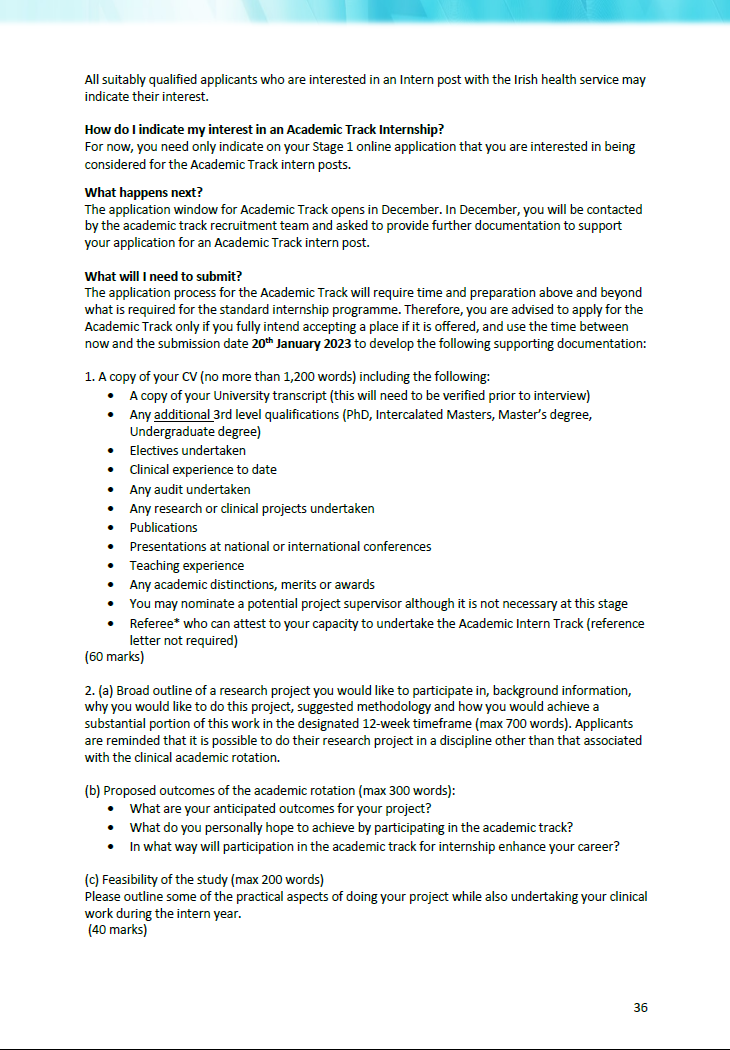


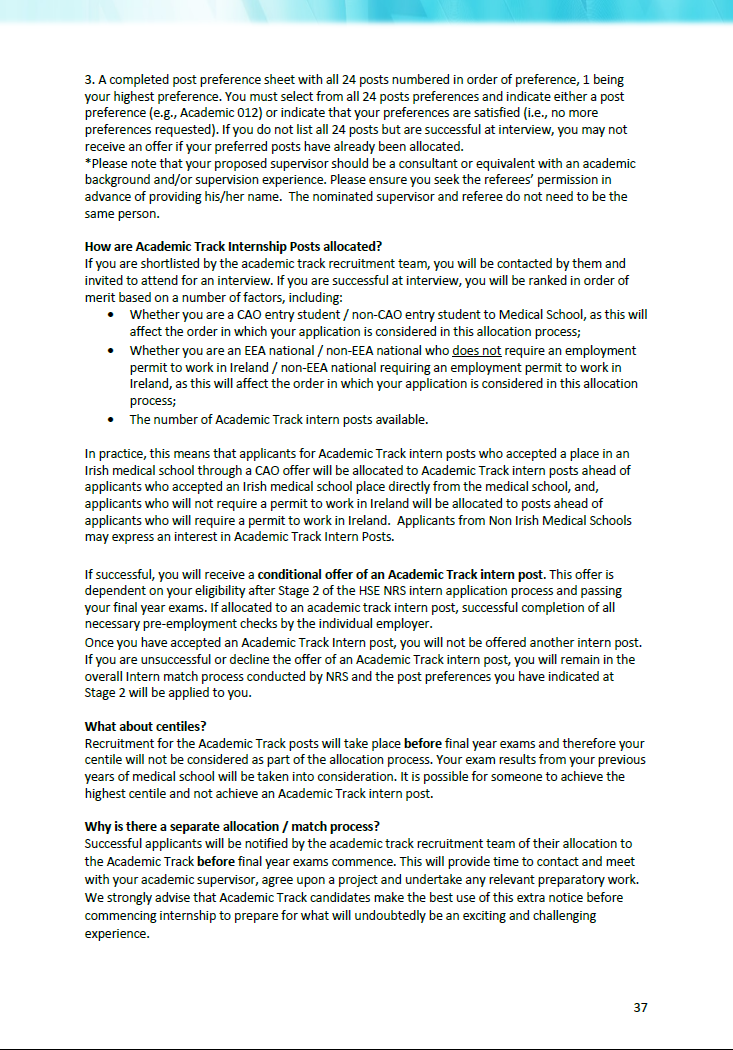

Supplement: Supplementary file 1 — Additional file 1. [file 12909_2023_4192_MOESM1_ESM.docx]
