# Supplementary material for: Gender bias in academic medicine: a resumé study
Source: BMC Med Educ. 2023 May 1;23:291. doi: 10.1186/s12909-023-04192-6 (PMC10152728; doi:10.1186/s12909-023-04192-6)

# Supplemental File 2: Distributions of scores

## Distribution of non-anonymous scores


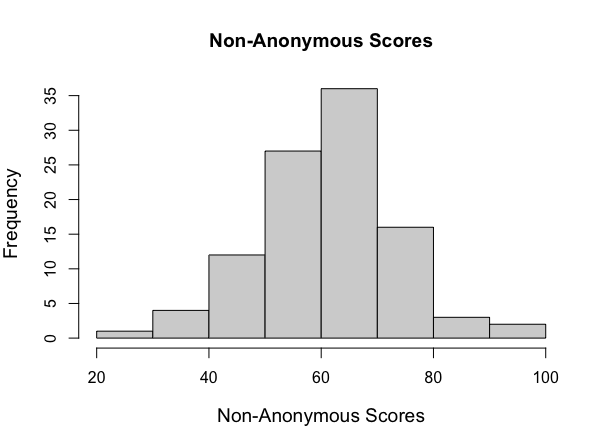


## Distribution of anonymous scores


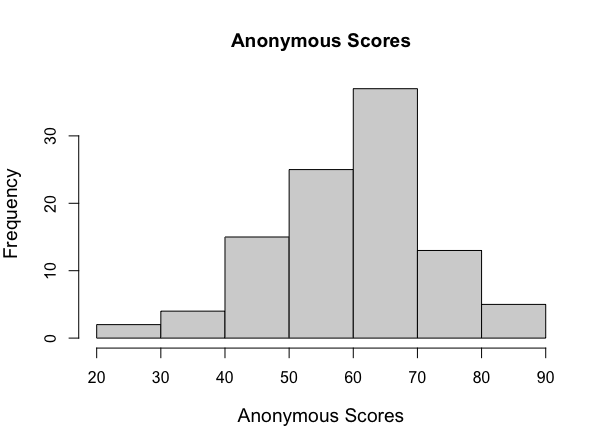


## Distribution of averages of anonymous and non-anonymous scores


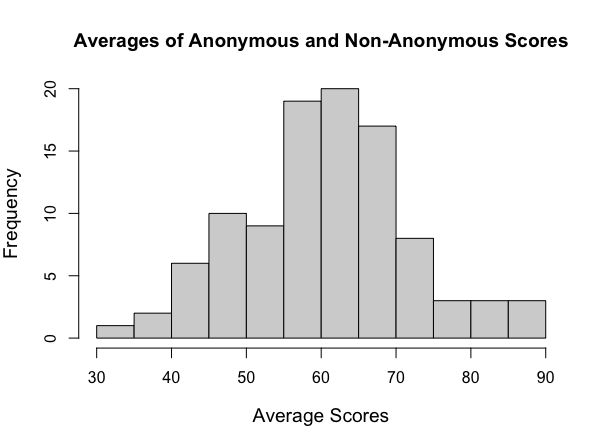

Supplement: Supplementary file 2 — Additional file 2. [file 12909_2023_4192_MOESM2_ESM.docx]
